# The association between the aggregate index of systemic inflammation and risk of rheumatoid arthritis: retrospective analysis of NHANES 1999–2018

**DOI:** 10.3389/fmed.2024.1446160

**Published:** 2024-08-22

**Authors:** Xiaoshuang Yin, Jinmei Zou, Jing Yang

**Affiliations:** Department of Immunology, Mianyang Central Hospital, School of Medicine, University of Electronic Science and Technology of China, Mianyang, Sichuan, China

**Keywords:** aggregate index of systemic inflammation, rheumatoid arthritis, NHANES, biomarker, risk factor

## Abstract

**Objective:**

The investigation purpose was to examine the correlation between the aggregate index of systemic inflammation (AISI) and rheumatoid arthritis (RA) by utilizing the NHANES database from the years 1999 to 2018.

**Methods:**

The NHANES database was utilized to extract data spanning from 1999 to 2018. AISI, comprising neutrophils (NEU), monocytes (MONO), platelets (PLT), and lymphocytes (LYM), was computed based on counts. The identification of RA patients was accomplished through questionnaire data. To investigate the connection between AISI and RA, a weighted multivariate regression and subgroup analysis were conducted. In addition, restricted cubic splines (RCS) were employed for examining non-linear associations.

**Results:**

The study encompassed a total of 41,986 patients, among whom 2,642 (6.29%) were diagnosed with RA. Upon controlling for all covariates, the outcomes of the multivariate logistic regression assay demonstrated a statistically significant association between higher Ln(AISI) levels and elevated odds of RA (odds ratio [OR]: 1.097; 95% confidence interval [CI]: 1.096–1.099, *p* < 0.001). The interaction test findings indicate that there is no statistically significant impact within this particular association. The results of the RCS regression model revealed a non-linear pattern in the correlation between Ln(AISI) and RA. The threshold level of AISI for RA was determined as 298.9. The risk of RA rises steeply when AISI surpasses the threshold value.

**Conclusion:**

Overall, a positive association has been observed between AISI and RA. This study highlights the potential of AISI as an innovative, vital, and appropriate inflammatory biomarker for predicting the risk of developing rheumatoid arthritis in older individuals residing in the United States.

## Introduction

Rheumatoid arthritis (RA) is the most common immune system disorder that is distinguished with a chronic and systemic autoimmune inflammatory condition (1, 2). The disease manifests through three classic pathological presentations: chronic synovitis causing pannus formation, destruction of cartilage and bone, and joint deformities (3). RA imposes a significant economic and personal burden worldwide due to its high disability rate. Clinical manifestations can vary among patients, with additional systemic symptoms such as rheumatoid nodules, skin disease, eye involvement, lung disease, etc. (4). In spite of the considerable amount of research conducted, the etiology of RA is still complicated and not yet comprehensively elucidated. The primary cellular mechanisms contributing to the disease involve a dysregulation within osteoblasts and osteoclasts, exaggerated progression of synoviocytes, and dysfunction of immune cells (5). Inflammatory factors commonly implicated in RA involve tumor necrosis factor-α (TNF-α), IL-6, and IL-8, interleukin (IL)-17 (6, 7). Recent research has emphasized the crucial involvement of auto-reactive T cells in the development of RA (7). Naive CD4 T cells develop into pro-inflammatory helper T cells, which infiltrate tissues greater readily, triggering inflammation and death of immune cell (7–9).

Chronic inflammation is recognized as an early characteristic of numerous chronic diseases. Inflammation biomarkers encompass single parameters such various cytokines, acute inflammatory proteins, as C-reactive protein (CRP), and specific immune blood cells, including platelets (PLT), neutrophils (NEU), lymphocytes (LYM), and monocytes (MONO). Furthermore, two-parameter inflammation indices, such as the platelet-to-lymphocyte ratio (PLR), neutrophil-to-lymphocyte ratio (NLR), and monocyte-to-lymphocyte ratio (MLR), as well as three-parameter indices like those observed in systemic immune-inflammatory index (SII) and systemic inflammation response index (SIRI), were established. Existing evidence supports the notion that incorporating more parameters enhances the comprehensive reflection of the body’s inflammatory condition. A meta-analysis and systematic review demonstrated that the two-parameter inflammatory indices NLR and PLR could effectively differentiate between individuals suffering from RA exhibiting active disease and those without active disease (10). Liu et al. (11), in a study utilizing a large public database, investigated the connection among SII and the risk of RA and observed a positive association. As an innovative, vital, and appropriate inflammatory biomarker, SII has the potential to anticipate the risk of RA in the adult population of US. Additionally, a large multi-center clinical investigation provided evidence that another three-parameter inflammation index, SIRI, has the potentiality to function as a new, non-invasive indicator to facilitate the identification and prognostication of disease progression in RA, RA-associated interstitial lung disease (RA-ILD), and cancer development among individuals diagnosed with RA. Notably, SIRI exhibited superior performance compared to other two-parameter blood cell-based indices in assessing RA patients (12, 13).

The aggregate index of systemic inflammation (AISI), also known as the pan-immune-inflammation value (PIV), is a metric utilized to comprehensively evaluate the systemic inflammatory condition by analyzing complete blood counts (CBC). It provides an easily accessible measure and is calculated using a formula that incorporates specific immune system markers from the CBC. AISI has emerged as a new predictive indicator that has been investigated in individuals with conditions such as idiopathic pulmonary fibrosis (IPF) (14), COVID-19 (15–17), certain cancers (18–20), and hypertension (21). Studies have demonstrated its ability to differentiate individuals with IPF from normal individuals and its independent association with poor prognosis (14, 22). Additionally, AISI has shown predictive value for mortality in COVID-19 patients (15–17), as well as overall survival and progression-free survival in individuals suffering from cancer (18–20). Furthermore, the association between AISI and unfavorable prognosis has been observed to be statistically significant in individuals diagnosed with viral pneumonia (16). Due to its ability to comprehensively and consistently reflect the body’s inflammatory status through four parameters, AISI is widely utilized in clinical practice. However, thus far, no research has explored the relationship between AISI and the risk of developing RA. Hence, the objective of this research was to examine the potentiality of AISI as a prognostic tool for the risk of RA, utilizing information obtained from the National Health and Nutrition Examination Survey (NHANES) database.

## Methodology

### Data acquisition and research framework

The National Health and Nutrition Examination Survey (NHANES) is a meticulously designed cross-sectional investigation that focuses on gathering comprehensive data related to the medical and nutritional condition of households across the US. The database utilizes a sophisticated categorized, multistage cluster sampling methodology to guarantee a statistically sound sample of the entire population of the US (23). Our research employed NHANES database information, which conducts annual surveys involving approximately 5,000 patients nationwide. The database includes a wide range of information such as demographic details, dietary records, results of physical examinations, questionnaire responses, laboratory data, and restricted data access. NHANES was carried out across a period of 10 cycles, commencing from 1999 and concluding in 2018. The present study obtained ethical approval from the Research Ethics Review Board of the National Center for Health Statistics, and all subjects granted informed consent by affixing their signature to a consent form. Detailed information regarding the publicly accessible NHANES study design and data can be obtained at https://www.cdc.gov/nchs/nhanes/.

**Figure 1:**
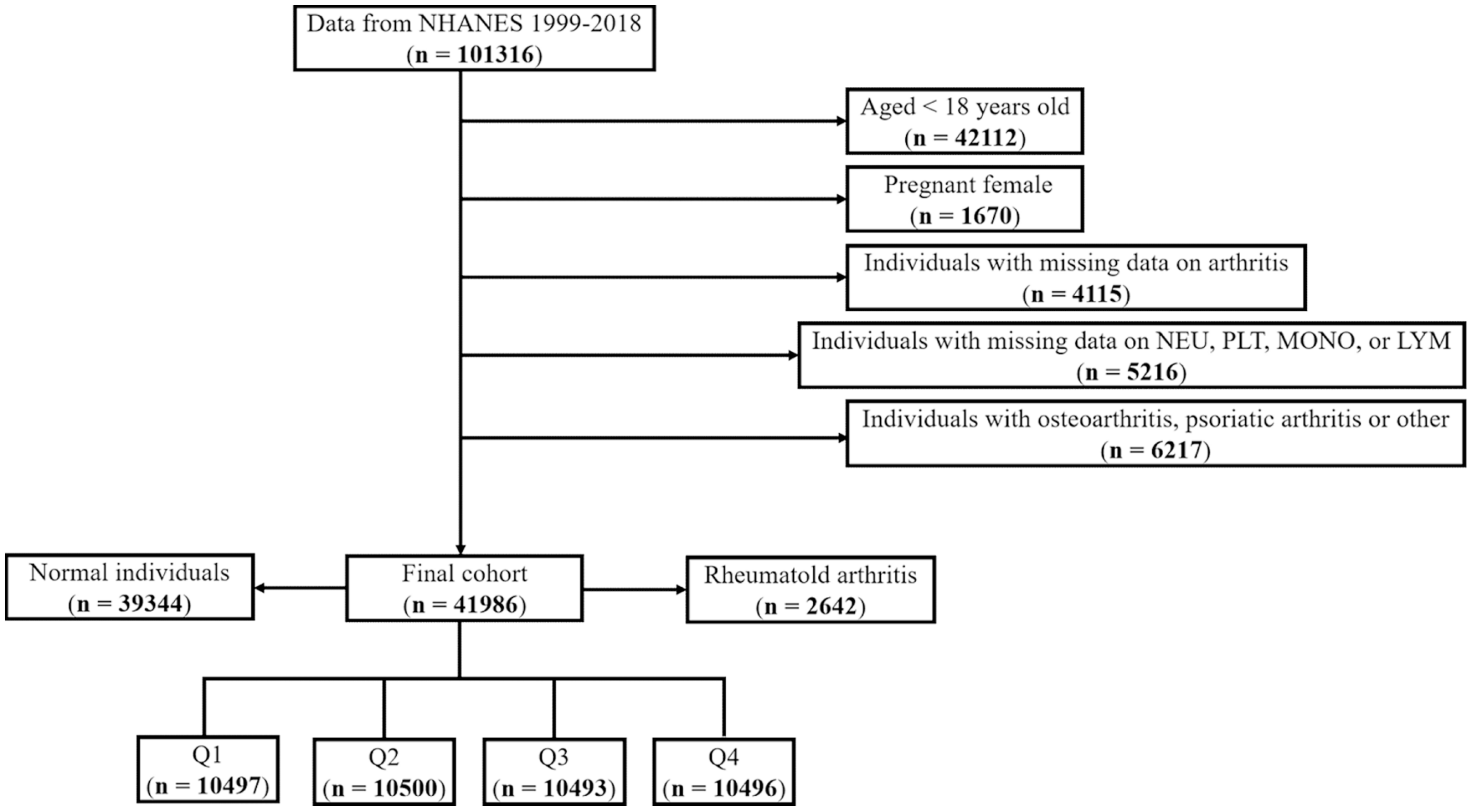
Flowchart for the selection process of individual from NHANES database of 1999–2018.

In this investigation, we applied the following exclusion criteria: (i) adults aged 18 years and above; (ii) pregnant females; (iii) individuals with incomplete data pertaining to arthritis; (iv) patients exhibiting missing data on NEU, PLT, MON, and LYM (Figure 1). Ultimately, a total number of 41,986 patients have been contributed to this analysis. As our study incorporated hematological parameters, the Mobile Examination Centers (MEC) weights were employed for the purpose of data analysis. The weight computation formula for the years 1999–2000 and 2001–2002 was 2/10 × wtmec4yr, while for the years 2003–2018, the formula was 1/10 × wtmec2yr.

### Aggregate index of systemic inflammation definition

**Figure 2:**
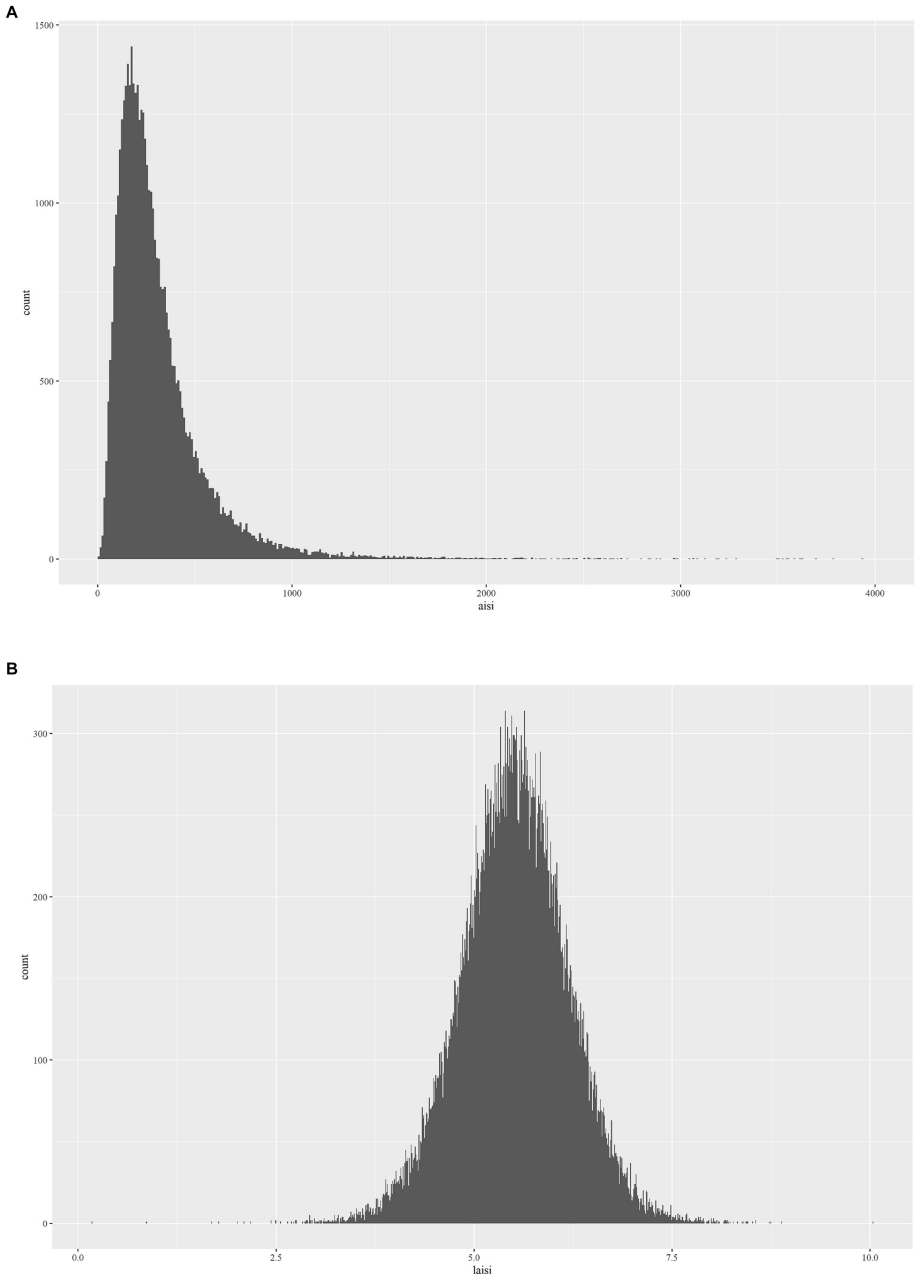
The distribution of **(A)** AISI and **(B)** Ln(AISI).

The Aggregate Index of Systemic Inflammation (AISI) is computed based on the enumeration and sizing approach of Beckman Coulter, utilizing parameters obtained from a complete blood count (CBC). This methodology involves automated sample processing, including dilution and mixing, as well as hemoglobinometry via employment of a single beam photometer. The white blood cell (WBC) differential utilizes VCS technology. In the context of NHANES Mobile Examination Center (MEC), the Beckman Coulter DxH 800 device conducts CBC analysis on blood samples and furnishes a blood cell distribution for all subjects. The computation formula for AISI, based on previous research findings, is (NEU * PLT * MONO)/LYM (24). In addition, during regression analysis (Figure 2B), AISI underwent an Ln-transformation due to the right-skewed distribution of these inflammatory markers (Figure 2A).

### Rheumatoid arthritis definition

The participants’ arthritis diagnosis was determined via a self-report questionnaire (MCQ160a) that was administered to them. The participants were surveyed to discover if they had ever received a medical diagnosis of arthritis from a healthcare professional. The available response alternatives were limited to a binary choice of either affirming or negating. Regarding rheumatoid arthritis, the individuals were additionally requested to specify the particular type of arthritis that had been diagnosed. The response options included “RA,” “Osteoarthritis,” “Psoriatic arthritis,” “Other,” “Refused,” and “Do not know.” An earlier investigation exhibited a high level of agreement (85%) within those diagnosed with self-reported arthritis and clinically validated arthritis (25).

### Data extraction

For our study, we extracted data on various factors including gender, age, ethnicity, educational attainment, body mass index (BMI), poverty-to-income ratio (PIR), work activity, smoking habits, alcohol consumption, hypertension, hyperglycemia, diabetes, and complete blood count parameters: WBC, RBC, NEU, PLT, MONO, and LYM.

### Statistical methodology

The R software (version 4.2.2, http://www.R-project.org) was employed for conducting the statistical analyses. Considering the complex, multi-stage sampling design of the NHANES database, we utilized MEC exam weights (wtmec4yr and wtmec2yr) for the assessment. The study employed weighted means and standard deviations (SD) to report the continuous variables, whereas weighted percentages were used for representing categorical variables. The chi-square test was utilized to conduct a comparison of categorical variables, while the t-test was utilized to compare continuous variables across distinct groups.

We observed that the distribution of AISI data was non-uniform and a significant positive skew. Thus, before performing statistical analysis, we performed a transformation of the AISI values. The present study employed weighted multivariate logistic regression models for investigating the correlation between RA and AISI. In the context of the crude model, there were no covariate adjustments. Model 1 underwent adjustment for gender, age, and ethnicity. Model 2 adjusted for age, gender, ethnicity, PIR, education, and BMI. Model 3 further set for gender, age, ethnicity, PIR, education, BMI, diabetes, hyperlipidemia, hypertension, alcohol consumption, smoking status, work condition, WBC, and RBC.

Additionally, we considered AISI as a categorical variable based on quartiles. To explore non-linear relationships, we employed restricted cubic splines (RCS). The current investigation employed generalized additive models and smooth curve fitting to examine the threshold effect of Ln(AISI) on the risk of RA and to identify the point of inflection.

Furthermore, we conducted interaction analyses and stratification by age, PIR, BMI, ethnicity, education, diabetes, hyperlipidemia, hypertension, work condition, alcohol consumption, and smoking status. All statistical tests exhibited two-sided, and a *p*-value <0.05 was considered statistically significant.

### Demographic and clinical features of the investigation cohort

The research included a total of 41,986 individuals, with 21,473 (50.6%) males and 20,513 (49.6%) females. Among the participants, 12,027 (19.4%) were aged above 60 years, and 17,588 (66.6%) identified as white ethnicity. Table 1 indicates the clinical features of the individuals stratified by AISI quartile. Statistically significant variations have been detected in gender, age, ethnicity, education, body mass index (BMI), poverty-to-income ratio (PIR), hypertension, diabetes, hyperglycemia, alcohol consumption, smoking status, work condition, and laboratory parameters (WBC, RBC, NEU, MONO, PLT, LYM, and AISI) (all *p* < 0.001).

**Table 1:** Weighed the demographic features of each individual.

Variables	Total	Q1	Q2	Q3	Q4	*p*-value
**Age**						<0.001
Below 60	29,959 (80.6%)	7,810 (83.0%)	7,612 (82.2%)	7,572 (80.8%)	6,965 (76.7%)	
Over 60	12,027 (19.4%)	2,687 (17.0%)	2,888 (17.8%)	2,921 (19.2%)	3,531 (23.3%)	
**Gender**						<0.001
Male	21,473 (50.6%)	5,278 (49.9%)	5,326 (50.6%)	5,339 (50.9%)	5,530 (51.0%)	
Female	20,513 (49.4%)	5,219 (50.1%)	5,174 (49.4%)	5,154 (49.1%)	4,966 (49.0%)	
**Race**						<0.001
White	17,588 (66.6%)	2,979 (54.9%)	4,286 (66.9%)	4,811 (69.6%)	5,512 (73.3%)	
Black	8,895 (11.3%)	3,622 (20.6%)	2,143 (10.7%)	1706 (8.5%)	1,424 (6.8%)	
Mexican American	7,854 (8.9%)	1713 (9.0%)	2090 (9.2%)	2,110 (9.1%)	1941 (8.4%)	
Other	7,649 (13.1%)	2,183 (15.5%)	1981 (13.1%)	1866 (12.8%)	1,619 (11.6%)	
**Education**						<0.001
Under high school	11,658 (17.7%)	2,868 (18.1%)	2,939 (17.1%)	2,904 (17.1%)	2,947 (18.4%)	
High school or equivalent	9,671 (24.0%)	2,238 (21.4%)	2,323 (22.7%)	2,454 (24.5%)	2,656 (27.2%)	
College graduate or above	20,657 (58.3%)	5,391 (60.5%)	5,238 (60.3%)	5,135 (58.4%)	4,893 (54.4%)	
**PIR**						<0.001
Below 1.3	15,754 (27.5%)	3,931 (27.8%)	3,866 (26.2%)	3,939 (27.5%)	4,018 (28.3%)	
1.3–3.5	14,435 (33.0%)	3,545 (32.5%)	3,561 (32.5%)	3,618 (32.5%)	3,711 (34.4)	
Over 3.5	11,797 (39.6%)	3,021 (39.7%)	3,073 (41.4%)	2,936 (40.0%)	2,767 (37.3%)	
**BMI**						<0.001
Below 25	12,762 (32.6%)	3,644 (38.7%)	3,219 (33.3%)	2,966 (29.9%)	2,933 (29.3%)	
25–30	14,090 (33.7%)	3,594 (34.2%)	3,649 (35.5%)	3,543 (33.9%)	3,302 (31.5%)	
Over 30	14,434 (33.7%)	3,118 (27.1%)	3,497 (31.2%)	3,809 (36.2%)	4,010 (39.2%)	
**Hypertension**						<0.001
No	25,429 (66.2%)	6,719 (70.8%)	6,552 (69.0%)	6,386 (66.0%)	5,772 (60.0%)	
Yes	16,557 (33.8%)	3,778 (29.2%)	3,948 (31.0%)	4,107 (34.0%)	4,724 (40.0%)	
**Diabetes**						<0.001
No	32,145 (81.8%)	8,192 (83.1%)	6,122 (83.4%)	8,055 (81.6%)	7,776 (79.4%)	
Pre-diabetes	2,886 (6.5%)	708 (6.5%)	742 (6.5%)	712 (6.7%)	724 (6.4%)	
Yes	6,955 (11.7%)	1,597 (10.4%)	1,636 (10.2%)	1726 (11.7%)	1996 (14.2%)	
**Hyperlipidemia**						<0.001
No	12,509(30.9%)	3,598(36.3%)	3,157(31.7%)	2,951 (29.2%)	2,803 (27.4%)	
Yes	29,477(69.1%)	6,899(63.7%)	7,343(68.3%)	7,542 (70.9%)	7,693 (72.6%)	
**Alcohol use**						<0.001
Never	9,752 (19.3%)	2,656 (21.9%)	2,473 (18.9%)	2,333 (18.2%)	2,290 (18.4%)	
Former	6,369 (12.6%)	1,542 (12.0%)	1,464 (11.3%)	1,536 (12.3%)	1827 (14.5%)	
Mild	12,250 (31.9%)	3,181 (32.8%)	3,169 (33.7%)	3,068 (32.4%)	2,832 (28.9%)	
Moderate	5,640 (15.6%)	1,429 (15.8%)	1,503 (16.9%)	1,395 (15.2%)	1,313 (14.6%)	
Heavy	7,975 (20.7%)	1,689 (17.5%)	1891 (19.2%)	2,161 (21.9%)	2,234 (23.7%)	
**Smoke**						<0.001
Never	23,112 (54.6%)	6,387 (60.9%)	6,044 (57.3%)	5,628 (53.5%)	5,053 (47.9%)	
Former	9,827 (23.3%)	2,257 (21.7%)	2,441 (23.6%)	2,482 (23.4%)	2,647 (24.1%)	
Now	9,047 (22.1%)	1853 (17.4%)	2015 (19.1%)	2,382 (23.1%)	2,796 (28.0%)	
**Work activity**						<0.001
No	15,069 (38.4%)	3,036 (30.5%)	3,665 (36.9%)	4,051 (41.1%)	4,317 (43.7%)	
Yes	26,917 (61.6%)	7,461 (69.5%)	6,835 (63.1%)	6,442 (58.9%)	6,179 (56.4%)	
**Laboratory parameters**						
WBC	7.2 (2.5)	5.7 (3.2)	6.6 (1.5)	7.5 (1.6)	9.0 (2.2)	<0.001
RBC	4.7 (0.5)	4.7 (0.5)	4.7 (0.5)	4.8 (0.5)	4.8 (0.5)	<0.001
NEU	4.3 (1.7)	2.8 (0.9)	3.7 (0.9)	4.5 (1.1)	5.9 (1.8)	<0.001
MONO	0.6 (0.2)	0.4 (0.2)	0.5 (0.1)	0.6 (0.1)	0.7 (0.2)	<0.001
PLT	254.2 (65.6)	213.7 (52.1)	240.6 (51.4)	261.8 (56.6)	293.8 (71.5)	<0.001
LYM	2.1 (1.4)	2.3 (2.7)	2.1 (0.8)	2.2 (0.7)	2.1 (0.7)	<0.001
AISI	320.7 (260.6)	114.3 (32.1)	203.2 (25.0)	307.2 (38.3)	619.9 (338.2)	<0.001

PIR, poverty-to-income ratio; WBC, white blood cell; RBC, red blood cell; BMI, body mass index; NEU, neutrophils; MONO, monocytes; PLT, platelets; LYM, lymphocytes; AISI, aggregate index of systemic inflammation.

**Figure 3:**
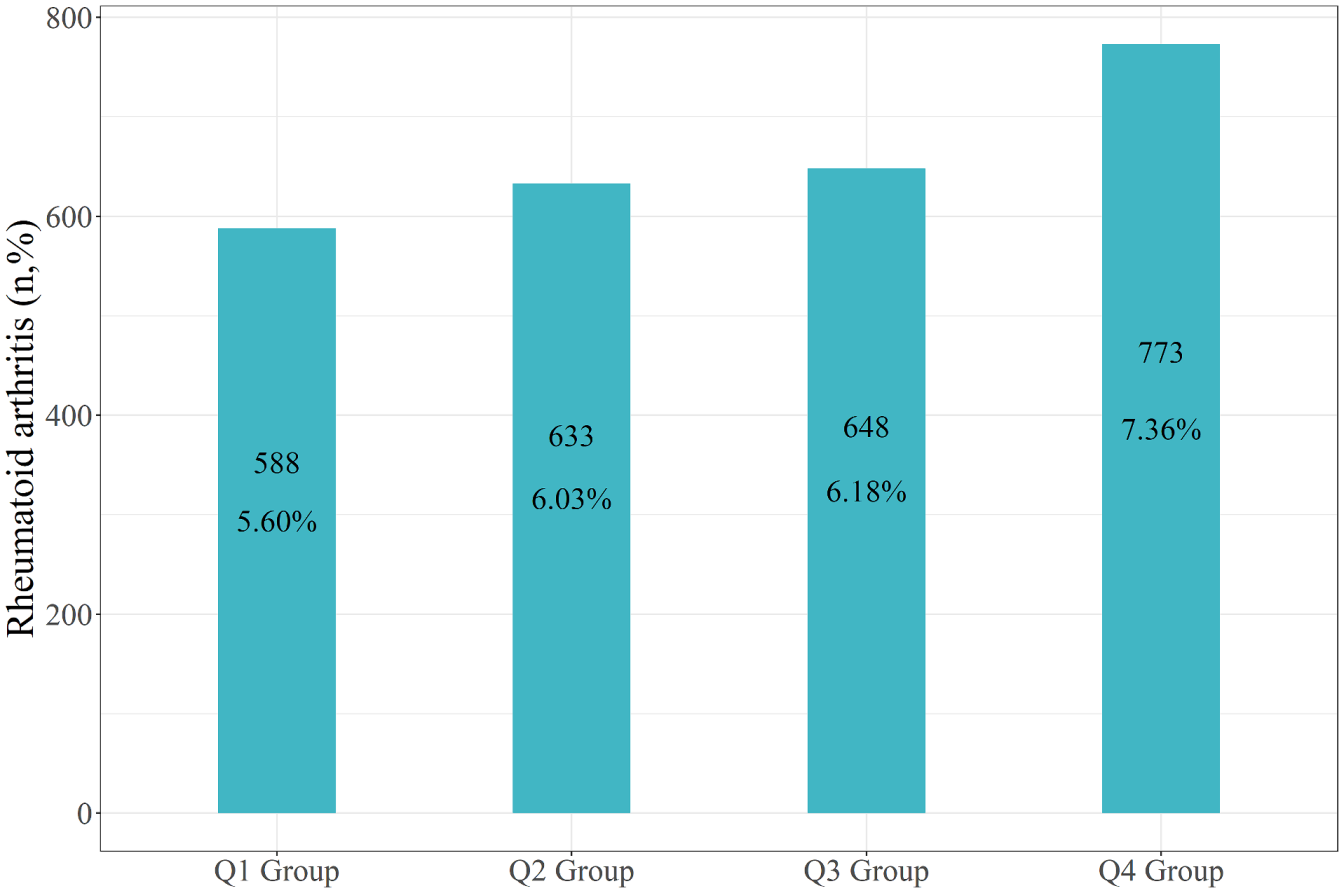
Number and percentage of RA in each group.

In the entire study population, the recorded incidence of rheumatoid arthritis (RA) was 2,642 individuals, accounting for 6.29% of the total participants. Specifically, there were 588 RA patients in the Q1 group (5.60%), 633 in the Q2 group (6.03%), 648 within the Q3 group (6.18%), and 773 within the Q4 group (7.36%) (Figure 3).

### RA univariate logistic regression analysis

According to Table 2, several factors were connected to an elevated risk of RA. The previous factors include advanced age (>60 years), being female, black ethnicity, elevated BMI (>25), smoking habits, hypertension, hyperglycemia, diabetes, former alcohol use, and higher levels of AISI. In contrast, individuals of other ethnicities who were Mexican-American, had a higher education, poverty-to-income ratio (PIR) > 1.3, alcohol consumers, and engaged in occupational activity exhibited a decreased level of being susceptible to RA. The odds ratios (OR) for these associations were higher than 1 (demonstrating elevated risk) or less than 1 (demonstrating reduced risk), with *p*-values less than 0.001, indicating statistical significance.

**Table 2:** RA weighted univariate logistic analysis.

Variables	OR (95% CI)	*p*-value
**Age**		
Under 60	Reference	
Above 60	3.511 (3.506–3.516)	<0.001
**Gender**		
Male	Reference	
Female	1.492 (1.490–1.494)	<0.001
**Race**		
White	Reference	
Black	1.496 (1.493–1.499)	<0.001
Mexican American	0.726 (0.724–0.728)	<0.001
Other	0.795 (0.793–0.797)	<0.001
**Education**		
Under high school	Reference	
High school or equivalent	0.761 (0.760–0.763)	<0.001
College graduate or above	0.501 (0.500–0.502)	<0.001
**PIR**		
Below 1.3	Reference	
1.3–3.5	0.781 (0.780–0.783)	<0.001
Over 3.5	0.545 (0.544–0.546)	<0.001
**BMI**		
Below 25	Reference	
25–30	1.191 (1.189–1.194)	<0.001
Over 30	1.867 (1.864–1.871)	<0.001
**Hypertension**		
No	Reference	
Yes	3.250 (3.245–3.254)	<0.001
**Diabetes**		
No	Reference	
Pre-diabetes	1.624 (1.620–1.629)	<0.001
Yes	2.602 (2.598–2.606)	<0.001
**Hyperlipidemia**		
No	Reference	
Yes	1.905(1.902–1.908)	<0.001
**Alcohol use**		
Never	Reference	
Former	1.845 (1.850–1.858)	<0.001
Mild	0.921 (0.921–0.923)	<0.001
Moderate	0.727 (0.725–0.729)	<0.001
Heavy	0.678 (0.676–0.679)	<0.001
**Smoke**		
Never	Reference	
Former	1.846 (1.843–1.849)	<0.001
Now	1.602 (1.600–1.605)	<0.001
**Work activity**		
No	Reference	
Yes	0.919 (0.917–0.920)	<0.001
**Aggregate index of laboratory parameters**		
AISI	1.202 (1.201–1.203)	<0.001

OR, odds ratio; PIR, poverty-to-income ratio; CI, confidence interval; BMI, body mass index; AISI, aggregate index of systemic inflammation.

### Correlation between RA and AISI

The outcomes of a weighted multivariate logistic regression analysis are presented in Table 3 which demonstrate a significant association among increased levels of AISI and an elevated risk of RA development. The association remained significant across different models. In the crude model, each unit increase in ln(AISI) value exhibited significant correlation with a 20.2% elevated risk of RA (OR: 1.202; 95% CI: 1.201–1.203, *p* < 0.001). Upon controlling for gender, age, and ethnicity in model 1, the risk remained elevated (OR: 1.199; 95% CI: 1.198–1.200, *p* < 0.001). Additional covariates may require further adjustment, including PIR, BMI, education, diabetes, hypertension, hyperlipidemia, alcohol consumption, smoking status, work condition, WBC, and RBC in model 3, still showed a favorable connection among AISI and RA risk (OR: 1.097; 95% CI: 1.096–1.099, *p* < 0.001). To assess the sensitivity of the results, AISI was also analyzed as a categorical variable (quartiles). Contrasted with those of the minimal quartile, the highest quartile showed a 30.8% increased risk of RA development in the crude model (OR: 1.308; 95% CI: 1.305–1.310, *p* < 0.001). This increased risk persisted in model 1 (OR: 1.314; 95% CI: 1.311–1.316, *p* < 0.001) and model 2 (OR: 1.179; 95% CI: 1.176–1.181, *p* < 0.001), although the magnitude of the correlation decreased slightly. In the context of model 3 which has been fully adjusted, the greatest quartile still had an 8.9% elevated risk of RA contrasted with the minimal quartile (OR: 1.089; 95% CI: 1.086–1.091, *p* < 0.001). These findings indicate that greater levels of AISI exhibited a positive correlation with an elevated risk of RA development, indicating the prospective effectiveness of AISI as an indicator for predicting RA risk.

**Table 3:** Weighted multivariate logistic analysis.

Variable	Crude model		Model 1		Model 2		Model 3	
	OR (95% CI)	*p*-value	OR (95% CI)	*p*-value	OR (95% CI)	*p*-value	OR (95% CI)	*p*-value
AISI	1.202 (1.201–1.203)	<0.001	1.199 (1.198–1.200)	<0.001	1.132 (1.131–1.133)	<0.001	1.097 (1.096–1.099)	<0.001
**Stratified by AISI quartiles**								
Q1	Ref.		Ref.		Ref.		Ref.	
Q2	1.118 (1.116–1.120)	<0.001	1.177 (1.174–1.179)	<0.001	1.131 (1.129–1.134)	<0.001	1.140 (1.138–1.143)	<0.001
Q3	1.044 (1.042–1.046)	<0.001	1.091 (1.089–1.093)	<0.001	1.008 (1.006–1.011)	<0.001	0.976 (0.974–0.978)	0.010
Q4	1.308 (1.305–1.310)	<0.001	1.314 (1.311–1.316)	<0.001	1.179 (1.176–1.181)	<0.001	1.089 (1.086–1.091)	<0.001
*P* for trend		<0.001		<0.001		<0.001		<0.001

Model 1: the variables of age, gender, and race were subjected to adjustment.Model 2: the variables of gender, age, race, PIR, education, and BMI were subjected to adjustment.

Model 3: the variables of gender, age, race, PIR, education, BMI, diabetes, hyperlipidemia, hypertension, alcohol consumption, smoke habits, work condition, WBC, and RBC were subjected to adjustment.

### Non-linear relationship between RA and AISI

**Figure 4:**
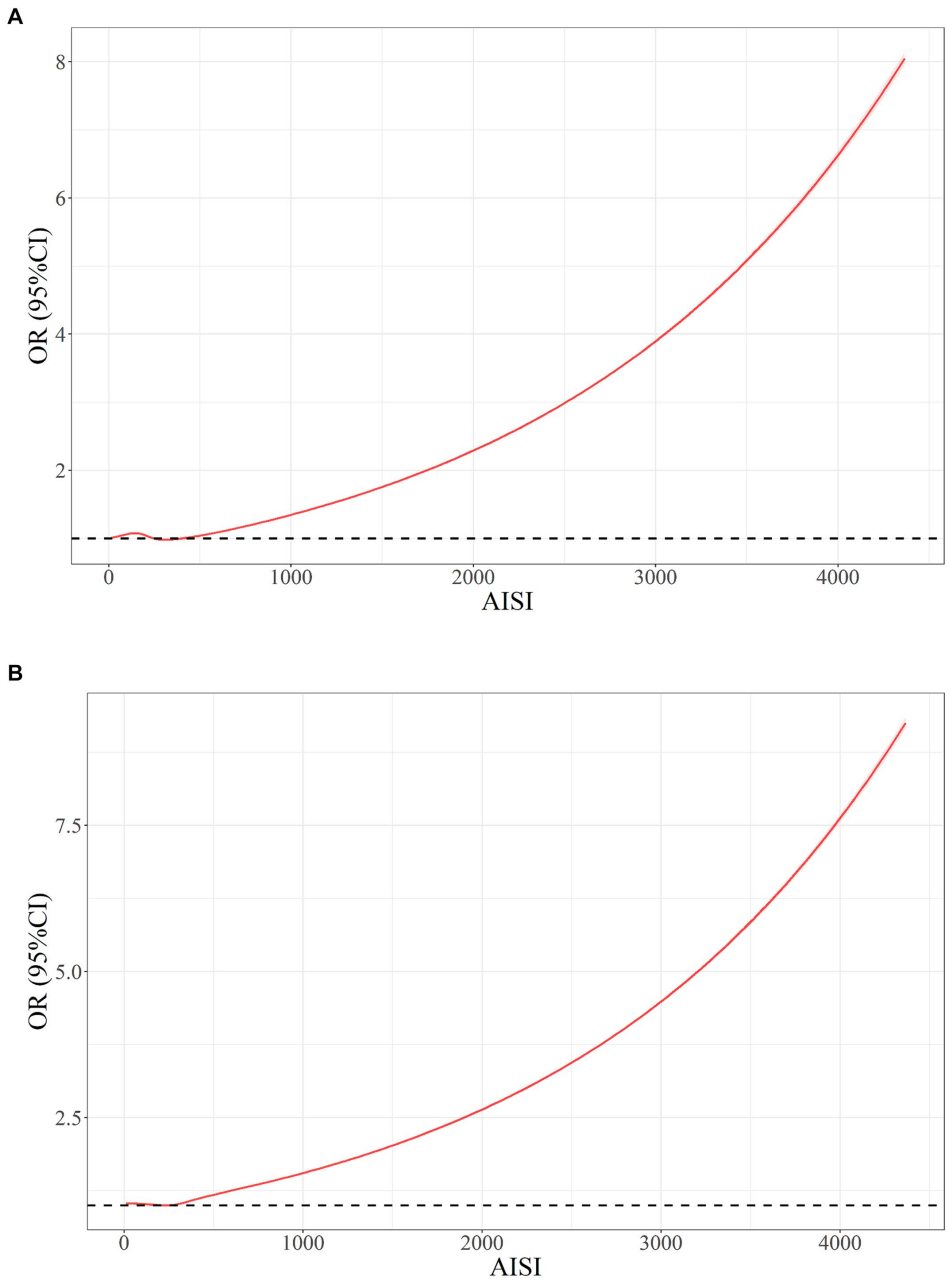
The non-adjusted connection among AISI and RA for **(A)** unadjusted and **(B)** full-adjusted.

The results obtained from restricted cubic splines (RCS) analysis revealed a non-linear correlation among ln(AISI) and RA risk within both the original model (Figure 4A) and the model was adjusted for multiple covariates (Figure 4B) (*p* < 0.001). The relationship exhibited a threshold effect, characterized by an inflection point at an ln(AISI) value of 5.70, corresponding to an AISI value of 298.9. Below this cutoff value, the risk of RA remained relatively constant. However, once the ln(AISI) value surpassed the cutoff point, the risk of RA escalated rapidly, demonstrating a J-shaped non-linear relationship.

### Interaction test and subgroup analysis

**Figure 5:**
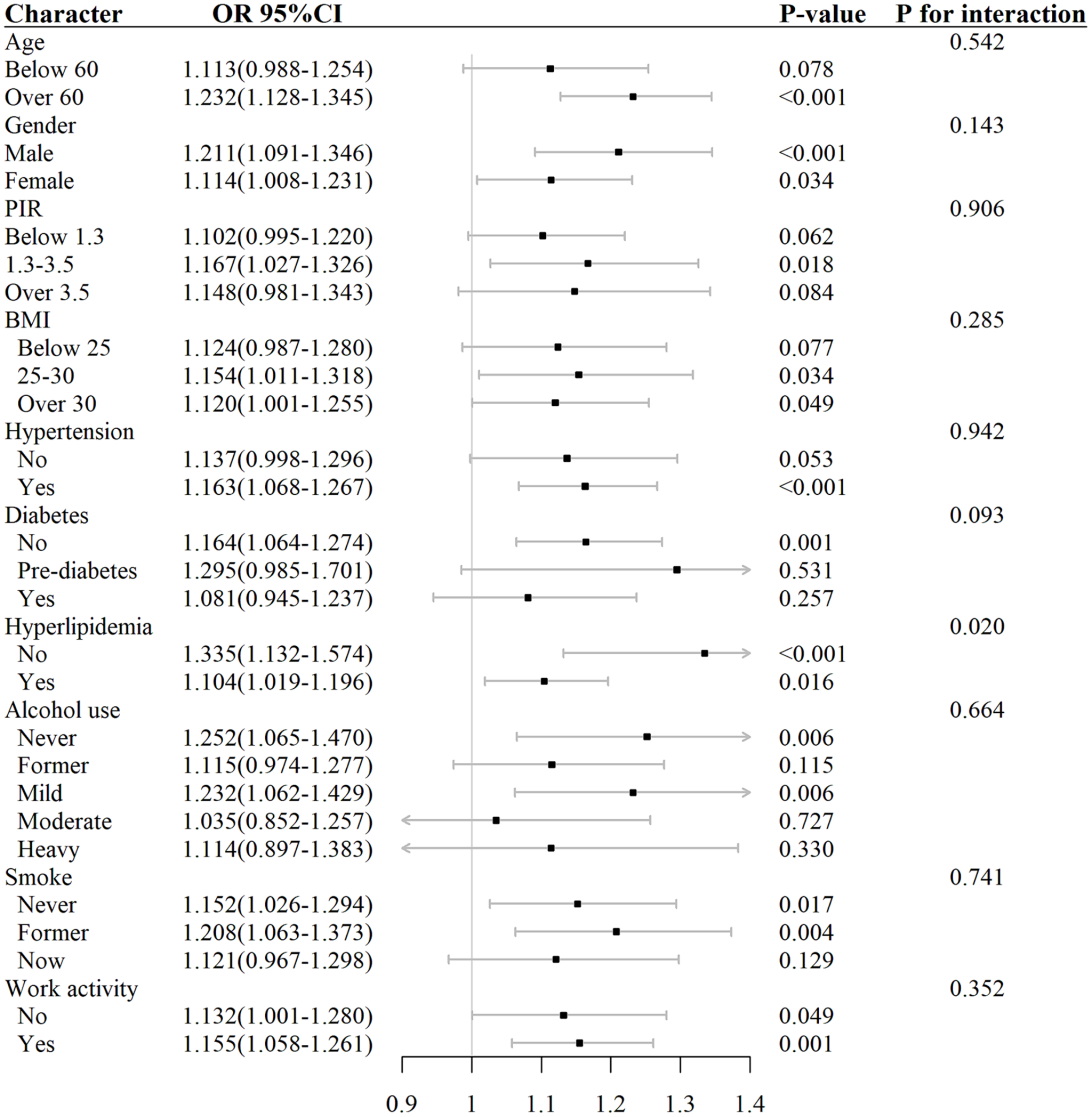
Forest plot for the correlation among AISI and RA.

The analysis of subgroups in Table 4 and Figure 5 showed that the connection among elevated AISI different levels and the risk of RA was not uniformly significant across all subgroups. Specifically, in subgroups such as age below 60, PIR greater than 3.5 or less than 1.3, BMI below 25, participants without hypertension, participants with diabetes, moderate and heavy alcohol consumption, and smokers, the correlation between AISI and RA risk did not demonstrate any statistically significant level (*p* > 0.05).

**Table 4:** Subgroup analysis for the correlation among AISI and RA.

Variable	OR (95% CI)	*p*-value	*P* for interaction
**Age**			0.542
Below 60	1.113 (0.988–1.254)	0.078	
Over 60	1.232 (1.128–1.345)	<0.001	
**Gender**			0.143
Male	1.211 (1.091–1.346)	<0.001	
Female	1.114 (1.008–1.231)	0.034	
**PIR**			0.906
Below 1.3	1.102 (0.995–1.220)	0.062	
1.3–3.5	1.167 (1.027–1.326)	0.018	
Over 3.5	1.148 (0.981–1.343)	0.084	
**BMI**			0.285
Below 25	1.124 (0.987–1.280)	0.077	
25–30	1.154 (1.011–1.318)	0.034	
Over 30	1.120 (1.001–1.255)	0.049	
**Hypertension**			0.942
No	1.137 (0.998–1.296)	0.053	
Yes	1.163 (1.068–1.267)	<0.001	
**Diabetes**			0.093
No	1.164 (1.064–1.274)	0.001	
Pre-diabetes	1.295 (0.985–1.701)	0.531	
Yes	1.081 (0.945–1.237)	0.257	
**Hyperlipidemia**			0.020
No	1.335 (1.132–1.574)	<0.001	
Yes	1.104 (1.019–1.196)	0.016	
**Alcohol use**			0.664
Never	1.252 (1.065–1.470)	0.006	
Former	1.115 (0.974–1.277)	0.115	
Mild	1.232 (1.062–1.429)	0.006	
Moderate	1.035 (0.852–1.257)	0.727	
Heavy	1.114 (0.897–1.383)	0.330	
**Smoke**			0.741
Never	1.152 (1.026–1.294)	0.017	
Former	1.208 (1.063–1.373)	0.004	
Now	1.121 (0.967–1.298)	0.129	
**Work activity**			0.352
No	1.132 (1.001–1.280)	0.049	
Yes	1.155 (1.058–1.261)	0.001	

Contrastingly, in subgroups such as age over 60, PIR between 1.3 and 3.5, BMI above 25, participants with hypertension, participants without diabetes or hyperlipidemia, mild alcohol consumption, smokers, and those engaged in work activity, the correlation between elevated AISI levels and the risk of RA exhibit statistically significant level (*p* < 0.05).

The outcomes of the interaction test (Table 4) indicated that factors such as age, gender, PIR, BMI, hypertension, diabetes, alcohol consumption, smoking habits, and work activity did not significantly influence the association between AISI and RA risk (interaction *p* > 0.05), except for hyperlipidemia, where the interaction was statistically significant (interaction *p* < 0.05).

## Discussion

In this study, a total of 41,986 participants from the NHANES 1999–2018 cohort were included, consisting of 21,473 males and 20,513 females. Among these participants, 2,642 individuals were diagnosed with RA. Comparing the AISI levels between healthy individuals and those with RA, it was observed that patients with RA had higher AISI levels. After adjusting for all covariates, a J-shaped non-linear correlation was discovered among AISI and the potential risk of RA. These findings suggest that there is a critical threshold value of AISI above which the risk of RA increases significantly. Below this threshold, the relationship between AISI and RA risk remains relatively stable. The inflection point was found to be at an AISI value of 289.9, indicating that when AISI exceeds this value, the risk of RA increases rapidly. Stratification analysis revealed that an association among AISI and RA risk may vary among different subgroups, with some subgroups showing a significant association while others do not. In certain subgroups, the association between elevated AISI levels and RA risk was not statistically significant, such as age below 60, those with PIR values greater than 3.5 or less than 1.3, BMI below 25, non-hypertension, history of diabetes, moderate and heavy alcohol consumers, and smokers. The interaction analysis indicates that most demographic and clinical factors do not modify the association, except for hyperlipidemia, which appears to have a modifying effect.

These findings highlight the potential association between AISI and the risk of RA, with the understanding that this relationship is influenced by various factors and may vary based on age, PIR, BMI, hypertension, diabetes, alcohol consumption, and smoking status. The study conducted by Liu et al. (11) investigated the relationship between the SII and the risk of RA, revealing a positive association. SII, as an innovative and vital inflammatory biomarker, has demonstrated potential in predicting the risk of RA within the adult population of the U.S. Building upon the work of Liu et al., we conducted an exploratory investigation into the association between a novel four-parameter inflammatory index, the AISI, and the risk of RA. Our findings were consistent with previous studies. Notably, AISI encompasses a broader range of inflammatory pathways, suggesting that it may have superior predictive value and efficacy compared to SII. However, further research is required to validate these theoretical advantages. It is worth noting that our study is the initial report to indicate increased AISI levels in individuals diagnosed with RA compared to healthy controls, utilizing the NHANES database. The AISI was originally introduced by Putzu et al. (24), who proposed the formula as the product of platelet count, monocyte count, and NLR. Subsequent studies have explored the clinical implications of AISI in different diseases, idiopathic pulmonary fibrosis, COVID-19, including non-small-cell lung cancer, acute coronary syndrome, and various cancers. The study revealed a significant correlation between AISI and prognosis at the 6-week mark following the initiation of nivolumab therapy in individuals suffered from non-small-cell lung cancer, outperforming NLR and PLR in predicting outcomes (24). Similarly, studies involving idiopathic pulmonary fibrosis patients demonstrated significant variations within different levels of AISI among individuals and healthy controls, with AISI independently associated with the presence of the disease and mortality (14, 22). The investigation of AISI in COVID-19 patients revealed its potential in predicting disease mortality, particularly in individuals with chronic renal failure (26, 27). Nevertheless, the predictive significance of AISI in cancer individuals suffered from COVID-19 is still inconclusive, as some studies did not find an association with poor prognosis (28, 29). Furthermore, the use of AISI has demonstrated potential as a prognostic tool for anticipating the disease severity and the risk of intensive care unit admission between patients suffered from COVID-19 (30). In the context of individuals suffered from acute coronary syndrome who have undergone percutaneous coronary intervention, increasing AISI tertiles were associated with elevated major adverse cardiovascular events risk, suggesting its potential as a laboratory marker for identifying high-risk patients (31). Additionally, AISI has been investigated as a tool for predicting bone mineral density in women who have passed menopause, as well as a prognostic marker in various cancers such as esophageal cancer and prostate cancer (19, 20, 32). Recently, AISI has also been identified as a potential predictor for hypertension and an increased risk of cardiovascular mortality (21). Given the limited existing studies on the association between AISI and RA, our research sheds light on this relationship. Moreover, researchers should be aware of the right-skewed non-normal distribution of AISI in the NHANES database and consider performing Ln-transformation before data analysis. The AISI serves as a measure of systemic inflammation within individuals diagnosed with RA and proves to exhibit a powerful predictive features in the context of RA risk in our study. Specifically, higher AISI values are related to an elevated risk of RA. The current investigation outcomes emphasize the significance of further exploring the function of AISI in the terms of RA. RA affects approximately 0.5 to 1% of adults in developed nations, with an annual incidence of about 5–50 new cases per 100,000 individuals (33). The disease primarily manifests during middle age, and its prevalence rate among women is 2.5 times that of males (34, 35). In 1990, RA accounted for 28,000 deaths, a number that rose to 38,000 mortalities in 2013 (36).

The RA is a persistent autoimmune condition that is distinguished by the presence of joint inflammation, resulting in symptoms such as pain, swelling, stiffness, and gradual joint deterioration. Additionally, RA has the potential to affect multiple organ systems, such as the eyes, skin, lungs, and cardiovascular system (35). The accurate cause of RA is yet to be determined, it is considered to involve complex interactions among genetic, environmental, and immunological factors. Genetic predisposition to RA development has been observed, with a particular HLA defects have been found to be linked with an elevated susceptibility. The etiology of RA has been linked to various environmental factors, including infections, trauma, and smoking (4). RA patients exhibit systemic inflammation and generate autoantibodies, which are indicative of their immune system’s functioning, resulting in immune cell activation and the production of cytokines that lead to joints arthritis and functional decline (37). Although the exact etiology of RA is yet to be fully elucidated, enhancing our comprehension of the fundamental pathophysiological mechanisms could potentially facilitate the identification of innovative and efficacious treatments for this incapacitating condition. The AISI serves as an indicator for assessing systemic inflammation in individuals. It is derived by multiplying the production of platelet count by the monocyte count and the NLR. Previous studies have demonstrated that this biomarker can be employed to prognosticate death individuals suffering from cancer (19, 20) and as an indicator of disease progression in specific conditions (21, 27).

The present investigation exhibits various advantages. Initially, this represents the first investigation to find out the connection among AISI and RA utilizing a large sample size from the NHANES database spanning from 1999 to 2018. Additionally, we utilized a weighted logistic regression model to account for the NHANES database complex sampling design, thereby enhancing the accuracy and reliability of our conclusions. Furthermore, prior to the analysis, we utilized Ln-transformation of AISI to guarantee a normal distribution. Lastly, we utilized RCS and smooth curve fitting techniques to examine the non-linear relationship and determine inflection points. Nevertheless, this investigation is not without restrictions. Firstly, some variables were obtained through questionnaires and self-reports, which are subject to potential biases. Moreover, since the NHANES database did not include certain classical inflammatory factors (e.g., TNF-α, IL-6, IL-10, etc.), we were unable to incorporate these relevant indicators to yield more comprehensive results. It is imperative for future researchers to continue investigating inflammatory markers in the context of RA. The present study is expected to establish a scientific basis for upcoming investigations. In subsequent research projects, we intend to incorporate supplementary classical inflammatory markers to investigate the correlation between RA and systemic immune-inflammatory indicators.

## Conclusion

In brief, this research offers significant perspectives on the correlation between AISI and RA. We observed a favorable association among AISI and the risk of RA, with a clinically significant cutoff value of 289.9. Our findings highlight the potential of AISI as a novel and convenient inflammatory marker for predicting the risk of RA in the US adults. The affordability and ease of computation are notable advantages of AISI. We anticipate that AISI will become a reliable indicator for evaluating the risk of RA. Notably, AISI serves as a valuable tool to evaluate the inflammatory condition of RA individuals and can serve as an early warning indicator for unfavorable prognosis. This study outcome has implications for the establishment of new therapeutic strategies targeting inflammation and immune damage. Nevertheless, additional randomized clinical trials are crucial for confirming the aforementioned results.

## Data Availability

The original contributions presented in the study are included in the article/supplementary material, further inquiries can be directed to the corresponding author.
